# Novel thermodynamic model for simulation of hydrogen/diesel fueled PCCI engine

**DOI:** 10.1016/j.heliyon.2025.e42140

**Published:** 2025-01-21

**Authors:** Ghasem Jalivar, Elaheh Neshat

**Affiliations:** Faculty of Mechanical Engineering, Sahand University of Technology, Sahand New Town, Tabriz, Iran

**Keywords:** Hydrogen/diesel dual fuel, Low-temperature combustion, PCCI engine, Thermodynamic simulation, Wiebe function

## Abstract

Utilizing hydrogen as an environmentally friendly fuel in LTC engines, which are ultra-low emission engines, enhances efficiency and improves combustion phase control. This addresses environmental challenges posed by fossil fuels. Effective control of the combustion phase in LTC engines, considering factors like fuel type, injection timing, and EGR percentage, requires real-time control-oriented models to optimize engine performance. However, CFD models face significant challenges in this area. This study introduces a thermodynamic-empirical model for simulating hydrogen/diesel dual-fuel PCCI combustion with minimal calibratable parameters. The model, developed in EES software, is validated using experimental data. The model's MFB curves, CA50, and IMEP values are compared with experimental results. Additionally, the model's results are compared with a previously validated CFD model's results for the 1.9L GM engine, including pressure, temperature, MFB curves, IMEP, and thermal efficiency values. Validation results show average errors of 0.25 CAD for CA50 and 0.043 bar for IMEP. Verification results indicate a highest percentage difference in peak pressure of 3.85 % and an average error in peak pressure timing of 0.6 CAD. The average error of CA50 between the presented model and the CFD model is 1.28 CAD, indicating the Wiebe function accurately simulates the PCCI combustion phase. The highest percentage difference in IMEP and the average error in thermal efficiency between the thermodynamic model and CFD results are 2.7 % and 2.41 %, respectively. The validation and verification results demonstrate the model's high accuracy and reliability, making it suitable for control applications of hydrogen/diesel dual-fuel PCCI combustion and a powerful tool for researching and developing hybrid systems, including PCCI engines, as environmentally friendly systems.

**Table:** Nomenclature

aTDC	After top dead center	LTHR	Low temperature heat release
ANN	Artificial neural networks	MFB	Mass fraction burn
CAD	Crank angle degrees	MVM	Mean value model
CA50	Crank angle of 50 % heat release	PCCI	Premixed charge compression ignition
CFD	Computational fluid dynamic	RCCI	Reactivity controlled compression ignition
CI	Compression ignition	SI	Spark ignition
CR	Compression ratio	SOC	Start of combustion
EOC	End of combustion	SOI	Start of injection
EGR	Exhaust gas recirculation	TDC	Top dead center
EVO	Exhaust valve opening	VCR	Variable compression ratio
GM	General Motors	VGT	Variable geometry turbocharger
HCCI	Homogeneous charge compression ignition	CO	Carbon monoxide
ID	Injection duration	CO2	Carbon dioxide
IMEP	Indicated mean effective pressure	HC	Hydrocarbons
IVC	Intake valve closure	O2	Oxygen molecule
LTC	Low temperature combustion	NOx	Oxides of nitrogen (NO + NO2)

## 1 Introduction

The depletion of fossil fuel reserves due to increasing energy demand, coupled with the environmental challenges arising from increased fossil fuel consumption, such as global warming and elevated pollutant levels, is well-documented. Internal combustion engines (ICEs) are one of the most significant consumers of fossil fuels, for which strict regulations have been implemented to reduce their emissions. Achieving emission standards and reducing fossil fuel consumption in ICEs has directed recent research towards reducing pollutants, increasing efficiency, and using alternative fuel sources [1]. One of the best alternative fuel sources for ICEs is hydrogen fuel. Hydrogen fuel is considered an environmentally friendly fuel because it does not produce pollutants or greenhouse effects [2]. The latest and most advanced solution for reducing emissions and increasing the efficiency of ICEs is low-temperature combustion (LTC). LTC engines have lower emissions, lower fuel consumption, and higher efficiency [3].

Hydrogen has recently become a highly attractive energy solution because it can be derived from renewable sources. In response to the potential of hydrogen as a clean energy source, the European Commission has crafted a new strategy centered on hydrogen. This plan seeks to advance hydrogen usage throughout Europe. As part of this effort, the European Clean Hydrogen Alliance was established. The alliance's objective is to facilitate the widespread implementation of hydrogen technologies and infrastructure by 2030 [4].

Hydrogen can be produced mainly through biochemical and thermochemical methods [5]. In the biochemical method, which encompasses several techniques, hydrogen is produced through the decomposition of biomass by microorganisms such as bacteria, archaea, and algae, without generating pollution. Although biohydrogen, derived from biochemical processes, is purer and economically, the commercial adoption of biohydrogen is currently constrained by supply-side challenges. However, the implementation of emerging techniques, such as machine learning algorithms, can substantially enhance the optimization of various operational parameters for large-scale biohydrogen production [6]. In the thermochemical method, biomass undergoes conversion into biofuels, gases, and chemicals through the application of heat or pressure. Gasification is a key thermochemical process for converting biomass and waste into energy [7]. This process not only generates hydrogen but also produces other valuable byproducts such as syngas on a large scale, which can be utilized for electricity generation or as a chemical feedstock [8]. This method produces CO₂ emissions however the cultivation of biomass absorbs CO₂, potentially offsetting emissions from gasification, particularly when integrated with carbon capture technologies [9]. Hydrogen produced via gasification can be used in a combined gasifier-ICE system, either independently or in conjunction with other components in CHP systems.

Numerous studies have investigated the use of hydrogen as either a primary fuel or an additive in conventional gasoline and diesel engines. The findings indicate that hydrogen enhances the performance of gasoline engines and reduces CO and HC emissions [10]. However, while hydrogen decreases exhaust emissions of carbon-based pollutants and soot, it also increases the risk of engine knocking and NOx emissions [11]. The diatomic nature of hydrogen molecules results in their dissociation at high temperatures, which significantly contributes to NOx formation [12]. Hydrogen, with its high lower heating value and rapid flame propagation across a wide range of temperatures, pressures, and air-to-fuel ratios [13], enhances energy conversion efficiency under lean operating conditions with optimized combustion phases. Additionally, hydrogen's high diffusivity facilitates quick mixing with incoming air, forming a homogeneous mixture. These features of hydrogen result in lower NOx emissions due to low-temperature combustion [14].

As mentioned, LTC, without requiring fundamental changes to conventional diesel engines, has the ability to significantly reduce NOx and soot emissions while maintaining the same efficiency as diesel engines [15]. LTC is primarily known as homogeneous charge compression ignition (HCCI), premixed charge compression ignition (PCCI), and reactivity-controlled compression ignition (RCCI) engines. In all three combustion modes, efforts are made to create a homogeneous or semi-homogeneous mixture within the combustion chamber to minimize soot formation. Additionally, exhaust gas recirculation (EGR) is employed to reduce NOx emissions by lowering the combustion chamber temperature [16,17].

In the PCCI combustion mode, the fuel-air mixture is semi-homogeneous, resulting in a stratified air-fuel mixture within the combustion chamber. Consequently, a substantial portion of the mixture undergoes premixed combustion, while the remainder combusts in a diffusion manner. This enhances the controllability of the combustion phase [2], extends the engine's operating range compared to the HCCI mode, and significantly reduces NOx and soot emissions [18].

While LTC combustion, similar to PCCI, presents a cost-effective solution for mitigating environmental challenges and addressing the depletion of fossil fuel resources, overcoming their inherent disadvantages is crucial for their industrial advancement. This necessitates a comprehensive understanding of the influence of input parameters such as EGR, intake air temperature and pressure, and injection timing on output parameters including IMEP, CA50, exhaust temperature, and emissions [19]. Engine simulation models are powerful tools for understanding these effects. Particularly for controlling the combustion phase of LTC engines, which is the most significant challenge to overcome the disadvantages of LTC and harness its benefits, proposing control-oriented models is crucial and has attracted significant attention in recent years [20].

In general, the simulation methods for LTC engines can be categorized into two primary groups: black-box models and physical models, which are respectively referred to as system identification-based modeling and physics-based modeling [21].

In black-box models, primarily derived from artificial neural networks (ANN), experimental data and statistical methods are utilized to establish relationships between the engine's input and output variables. Therefore, black-box models used for simulating LTC engines [22,23] can simulate engine behavior without requiring detailed knowledge of the engine's physical processes. However, this lack of physical insight means these methods struggle to accurately compensate for system dynamics change in response to input variations. Particularly, as the combustion process becomes more complex, their predictive accuracy decreases [24]. Nevertheless, Recent advancements in ANN methods and the integration of black-box models with physical models, known as gray-box models, have led to improved simulation accuracy and reduced computational costs [24].

Physics-based models are classified into three categories: three-dimensional computational fluid dynamics (3D-CFD) models [25,26], multi-zone quasi-dimensional models [27,28], one-zone zero-dimensional models [29,30], with the latter two being known as thermodynamic models.

3D-CFD models are highly effective tools for simulating various phenomena within the combustion chamber, including mass flow, temperature gradients, and turbulence. Additionally, they facilitate detailed combustion modeling using both detailed and reduced chemical kinetic mechanisms, enabling accurate prediction of LTC engine emissions [31,32]. However, compared to the other two models, these physics-based models are computationally expensive, making them unsuitable for engine control applications. They are more appropriate for engine design [33,34]. Nevertheless, these models can serve as reference models for developing simpler models intended for control purposes.

In multi-zone quasi-dimensional models used to simulate LTC engines, the combustion chamber is divided into several zones, each with its own characteristics [35,36]. Depending on the model's complexity, these zones can interact with each other. Consequently, these models yield more accurate results for maximum pressure, combustion duration, combustion efficiency, and emissions compared to single-zone models. Additionally, they are simpler and faster computationally than 3D-CFD models. However, dividing the combustion chamber into more than three zones and accounting for their interactions increases the complexity of multi-zone quasi-dimensional models, making them unsuitable for engine control applications.

As previously mentioned, controlling the combustion phase of LTC engines is critical for enhancing fuel efficiency and reducing emissions. Therefore, it is essential for models to meet control objectives with minimal computational cost and sufficient accuracy. Zero-dimensional single-zone models are highly effective for this purpose. Despite assumptions such as complete homogeneity of the mixture throughout the combustion chamber, which result in overestimations of peak pressure and NOx emissions, and underestimations of heat release, UHC, and CO emissions [37], these models can predict ignition delay, peak pressure, and engine performance with low computational cost and acceptable accuracy. Single-zone zero-dimensional models are divided into three categories: mean value models (MVM), Wiebe-based models, and reaction-based models. These models are known as control-oriented models and, depending on the need to increase modeling accuracy while reducing computation time, they can be developed into two-zone models (burned and unburned or mixed and unmixed zones). Additionally, there is the capability to use a combination of these models for better control purposes. single zone model has been considered in many studies to simulate LTC [21].

Shaver et al. developed a zero-dimensional physics-based model for controlling propane-fueled HCCI combustion. This model uses an integral Arrhenius rate expression to account for the significance of temperature and key species in the ignition process. Results showed that using the Arrhenius expression achieved the closest agreement between simulation and experimental data [38]. Yasar et al. investigated single-zone modeling of HCCI combustion using the Wiebe function. This study compared the performance of the standard single-Wiebe function with the double-Wiebe function. The results showed that the best agreement between experimental and simulation data was achieved using the double-Wiebe function [39]. Ravi et al. presented a thermodynamic model for controlling HCCI combustion with exhaust re-compression. In this study, the fueling rate and valve timing were considered as input parameters, while IMEP and CA50 were the output parameters. Using the Arrhenius integral expression, SOC was determined as a function of the IVC temperature [40]. Yamasaki et al. proposed a control-oriented model for PCCI engine simulation. This discrete-cycle model was developed for multiple injection PCCI engines. In this model, Livengood-Wu and Arrhenius integrals are used for the pilot and main injection parts, respectively. The results showed that the proposed model effectively simulates multiple injection PCCI combustion [34]. Namar and Jahanian used a single-zone model to simulate hydrogen HCCI combustion. In this study, a chemical kinetic mechanism was employed to simulate the engine, and the engine operation was also analyzed from the perspective of the second law of thermodynamics [41]. Xiao et al. used a single-zone model to simulate hydrogen combustion. The results showed that the model can be used across a wide range of operating conditions [42].

Given the comprehensive review of the significance of hydrogen fuel and the models developed for simulating PCCI engines in the technical literature, several key points emerge. CFD models, due to their complexity, require substantial computational resources and are not suitable for real-time control applications. Therefore, the development of single-zone models for control applications is essential, as these models provide rapid and accurate insights into critical parameters and combustion characteristics of PCCI engines. The proposed single-zone models have numerous parameters that must be calibrated using extensive experimental data, making them costly in terms of calibration. Most existing models in the literature focus on HCCI combustion, with limited studies presenting control-oriented simulation models for PCCI combustion. The models presented have been validated for other fuels, and low-temperature combustion with hydrogen fuel has not yet been modeled. This study aims to present a physics-based model for simulating hydrogen/diesel PCCI combustion that, while simple, has very few calibratable parameters and is suitable for control purposes. This model simulates hydrogen/diesel PCCI combustion with high accuracy and low computational cost and can be integrated with complex CFD models to reduce computational time. Integrating hydrogen fuel with low-emission PCCI engines for power generation is a highly attractive option. Particularly, using PCCI engines with hydrogen fuel in hybrid systems, such as gasifier-PCCI or CHP systems coupled with PCCI engines, is a very suitable option for environmentally friendly power generation. Therefore, the proposed thermodynamic model can accurately simulate hydrogen-fueled PCCI combustion in hybrid systems with low computational cost, significantly aiding the development of such systems, while the application of CFD models in this area remains very costly and sometimes impractical.

## 2 Model description

### 2.1 Modeling approach

In this section, a thermodynamic model is developed to simulate hydrogen/diesel dual-fuel PCCI combustion. The proposed model is a discrete crank-based model that simulates PCCI combustion from intake valve closure (IVC) to exhaust valve opening (EVO). In this model, the IVC to EVO duration is divided into several processes, including: compression stroke (IVC-SOI), ignition delay time (SOI-SOC), combustion period (SOC-EOC), and expansion stroke (EOC-EVO). The main equations are presented in section 2.2, and the sub-equations with detailed explanations are reported in the Appendix of this manuscript. The necessary assumptions for developing the model include the following [43]:

1. The presented model simulates the closed cycle of a PCCI engine from IVC to EVO.
2. The in-cylinder mixture composition is assumed to be constant and follows the ideal gas law.
3. The entire system is considered as a single region with uniform temperature, pressure, and concentration of compounds.
4. Gas leakage during the cycle is neglected.
5. Heat transfer rates in each process are estimated using a heat transfer expression to the cylinder walls, and radiative heat transfer is ignored.
6. The injected fuel is instantaneously mixed with the in-cylinder charge and also follows the ideal gas law.
7. The enthalpy of the injected fuel is neglected due to its insignificant value.
8. The properties of the fuel-air-EGR mixture are calculated considering the global combustion reaction of the fuel.

### 2.2 Extraction of equations

By dividing the closed interval between IVC and EVO into four processes, it becomes possible to describe each process thermodynamically. These descriptions are based on a physics-based model, which will be developed in this section, and the aforementioned assumptions.

#### 2.2.1 Intake valve closure conditions and in-cylinder composition

Given the intake manifold conditions ($T_{in}$ $,{\;P}_{in}$), the total input energy to the engine ($Q_{in,engine}$), and the pressure at IVC as input data, other thermodynamic properties at IVC can be determined. With different hydrogen energy rates, the mass of hydrogen and diesel fuel can be calculated using equation (1) [44].

(1) $$\alpha_{H_{2}}=\frac{m_{H_{2}}.{LHV}_{H_{2}}}{\underbrace{m_{H_{2}}.{LHV}_{H_{2}}+m_{diesel}.{LHV}_{diesel}}}$$

$$Q_{in,engine}$$

The total fuel mass is determined using equation (2).

(2) $$m_{f}{=m}_{H_{2}}+m_{diesel}$$

The air-EGR mixture density is determined by equation (3).

(3) $$\rho_{in}=\frac{P_{in}}{R_{g}T_{mix}}$$

where $R_{g}$ is the gas constant and $T_{mix}$ is the average temperature of the air and EGR mixture at the intake manifold, obtained using equation (4).

(4) $$T_{mix}=\left({1-\alpha }_{egr}\right).T_{in}+\alpha_{egr}{.T}_{egr}$$

where $\alpha_{egr}$ and ${.T}_{egr}$ represent the percentage and temperature of EGR, respectively.

By calculating the gas density, the air, EGR, and total intake mass values are determined using equations (5), (6), (7)).

(5) $$m_{air}=\left({1-\alpha }_{egr}\right).\rho_{in}.V_{d}$$

(6) $$m_{egr}{=\alpha }_{egr}.\rho_{in}.V_{d}$$

(7) $$m_{in}{=m}_{air}+m_{egr}+m_{H_{2}}$$

where $V_{d}$ is displacement volume. The IVC temperature is then obtained from equation (8).

(8) $$P_{ivc}.V_{ivc}=m_{in}.R_{g}{.T}_{ivc}$$

Where $V_{ivc}$ is the cylinder volume at IVC. The cylinder volume and surface area at each crank angle are obtained using equations (9), (10)) as slider-crank mechanism equations [45].

(9) $$V_{\theta }=V_{c}+\pi .\frac{B}{4}.(L+a_{l}-a_{l}.\cos\left(\theta \right)-\sqrt{L^{2}-({a_{l}.\sin\;\left(\theta \right))}^{2}}$$

(10) $$A_{\theta }=2.\pi .\frac{B^{2}}{4}+\pi .B.(L+a_{l}-\left(a_{l}.\cos\left(\theta \right)+\sqrt{L^{2}-({a_{l}.\sin\;\left(\theta \right))}^{2}}\;\right)$$

where B is the piston bore, L is the rod length, $a_{l}$ is the crank radius and $V_{c}$ is the clearance volume, as determined by equation (11).

(11) $$V_{c}=\frac{V_{d}}{r_{c}-1}$$

Where $r_{c}$ is the compression ratio.

Using the overall combustion reaction, the properties and in-cylinder composition can be determined. Considering hydrogen and diesel as fuels, the overall combustion reaction is written as equation (12):

(12) $$\emptyset \left(aC_{x}Hy+bH_{2}\right)+\left(a\left(x+y⁄4\right)+b/2\right)\left(O_{2}+3.76N_{2}\right)+\alpha_{\mathrm{e}gr}\left(\left(ax\phi CO_{2}+\left(\left(y⁄2\right)a+b\right)\phi H_{2}O+3.76\left(\left(x+y/4\right)a+b/2\right)\right)N_{2}+\left(\left(x+y/4\right)a+b/2\right)\left(1-\emptyset \right)O_{2}\right))\to d_{1}CO_{2}+d_{2}H_{2}O+d_{3}O_{2}+d_{4}N_{2}$$

Where ∅ is the equivalence ratio, and a and b are the volume fractions of hydrogen and diesel respectively. $d_{4}$ -$d_{1}$ represent the product moles obtained using atomic balance for different species.

#### 2.2.2 Polytropic compression

Initially, it is assumed that the compression process occurs isentropically for an ideal gas [45], so the temperature, pressure, and other thermodynamic properties at the start of injection (SOI) are obtained assuming an air composition. Then, the heat transfer to the cylinder walls is calculated using equation (13).

(13) $$Q_{compression}=h_{c}.A_{av}.\left(T_{av}-T_{wall}\right)$$

Where $A_{av}$ is the average cylinder surface area, $T_{av}$ is the average cylinder temperature, $T_{wall}$ is the cylinder wall temperature, and $h_{c}$ is the convection heat transfer coefficient, modeled using the modified Woschni equation as equation (14) [46].

(14) $$h_{c}=0.0034.{\left(H\right)}^{-0.2}.{\left(T_{av}\right)}^{-0.73}.{\left(P_{av}\right)}^{0.8}{\left(w\right)}^{0.8}$$

where H is the effective height of the combustion chamber ($A_{av}/$ $V_{av}$), $P_{av}$ is the average chamber pressure and w is the gas velocity characteristic.

The work done during the compression stroke is calculated using the energy equation, represented as equation (15).

(15) $$W_{compression}=U_{soi}{-U_{ivc}+Q}_{compression}$$

Where $U_{ivc}$ and $U_{soi}$ are the internal energies at IVC and SOI.

By calculating the work using the energy equation, the specific heat ratio for the compression process ($K_{c}$) is modified using the work equation for a polytropic process, as shown in equation (16). With this modification, instead of an isentropic process, the compression process is considered to be polytropic [45].

(16) $$K_{c}=1+\frac{P_{soi}.V_{soi}-P_{ivc}.V_{ivc}}{W_{compression}}$$

With $K_{c}$ obtained from the above equation, $T_{soi}$ and $P_{soi}$ values are modified using the following polytropic equations, and other thermodynamic properties are also adjusted.

(17) $$T_{soi}=T_{ivc}{.\left(\frac{V_{ivc}}{V_{soi}}\right)}^{K_{c}-1}$$

(18) $$P_{soi}=P_{ivc}{.\left(\frac{V_{ivc}}{V_{soi}}\right)}^{K_{c}}$$

After calculating $T_{soi}$ and $P_{soi}$ using equations (17), (18)), the solution process iterates through equations (13), (14), (15), (16)). During this process, $T_{soi}$ and $P_{soi}$ are recalculated, and this continues until the temperature difference between the current and previous iterations at the SOI falls below the specified error.

Incorporating the polytropic process into this simulation results in a deviation from idealized modeling. The polytropic exponent ($K_{c}$), which reflects the specific heat ratio and heat transfer, varies according to operating conditions and engine specifications [43]. The aforementioned correction method yields an optimal value for $K_{c}$ during the compression and expansion processes, facilitating precise modeling of pressure and temperature during compression, ignition delay, and expansion phases, as well as accurate prediction of the combustion phase. Furthermore, integrating the polytropic process with the cylinder wall heat transfer model significantly enhances the model's accuracy in predicting pressure, temperature, and heat transfer rates. This is a key feature of the presented model that distinguishes it from other models available in the literature.

#### 2.2.3 Ignition delay time

The time interval between fuel injection (SOI) and the start of combustion (SOC) is termed the ignition delay time. Accurate calculation of this period is crucial for simulating the combustion phase in an engine. Numerous ignition delay time equations have been proposed in the technical literature, among which the equation presented by Assanis [47] is considered most suitable for diesel engines. Alstine [48] extended Assanis' equation for diesel engines. In this study, Alstine's model is adopted with a modification to the activation energy term as (71.3/(CN+25)) to account for the cetane number of the dual fuel [49]. The ignition delay time are calculated using equation (19).

(19) $$ID=C_{id}.{\chi_{O_{2}}}^{-1.14}.{P_{m}}^{-0.51}\;\exp\left(2100.\frac{\frac{71.3}{{CN}_{mix}+25}}{T_{m}}\right).0.006.N$$

Where $C_{id}$ is the ignition delay coefficient, varying with engines and conditions [49]. $\chi_{O_{2}}$ is the oxygen mass fraction at the intake manifold, $T_{m}$ and $P_{m}$ are the temperature and pressure at SOI, and ${CN}_{mix}$ is the cetane number of the dual-fuel mixture, obtained using equation (20) [50].

(20) $${CN}_{mix}=y_{H_{2}}.{CN}_{H_{2}}+y_{diesel}.{CN}_{diesel}$$

$y_{H_{2}}$ and $y_{diesel}$ are the mass fractions of hydrogen and diesel, respectively.

The combustion start time and total mass inside the chamber before combustion are obtained from equations (21), (22)).

(21) $$\theta_{soc}=\theta_{soi}+ID$$

(22) $$m_{t}=m_{in}+m_{diesel}$$

Assuming a polytropic process, the pressure and temperature at SOC, and the work done in the SOI-SOC interval, are calculated from equations (23), (24), (25)).

(23) $$T_{soc}=T_{soi}{.\left(\frac{V_{soi}}{V_{soc}}\right)}^{K_{c}-1}$$

(24) $$P_{soc}=P_{soi}{.\left(\frac{V_{soi}}{V_{soc}}\right)}^{K_{c}}$$

(25) $$W_{soi-soc}=\left(1+\frac{P_{soi}.V_{soi}-P_{soc}.V_{soc}}{K_{c}-1}\right)$$

#### 2.2.4 Combustion stroke

Combustion equations are solved from the start of combustion (SOC) to the end of combustion (EOC) in steps of 1° CA. The Wiebe function is used to calculate the Mass Fraction Burned (MFB), as shown in equation (26) [51].

(26) $$x_{b,i}=1-\exp\;\left(-a^{\prime }.{\left(\frac{\theta -\theta_{soc}}{\theta_{d}}\right)}^{m^{\prime }+1}\right)$$

where i represents the time step, $\theta_{d}$ is the combustion period, and $a^{\prime }$ and $m^{\prime }$ are Weibe function parameters, reported in Appendix A.

In the combustion period, the value of heat transfer ($Q_{i}$) is obtained from equation (13), as in the compression stroke. The value of work done in each time step and the total work are obtained from equations (27), (28)).

(27) $$W_{combustion,i}=\frac{\left(P_{i}+P_{i+1}\right)}{2}.\left(V_{i}-V_{i-1}\right)$$

(28) $$W_{combustion,t,i}=W_{combustion,i-1}+W_{combustion,i}$$

The heat release rate at each time step is also obtained from equation (29).

(29) $$Q_{h,i}=\eta_{c}.m_{f}.\left(x_{b,i}-x_{b,i-1}\right).{LHV}_{mix}$$

$\eta_{c}$ is the combustion efficiency and ${LHV}_{mix}$ is the low heating value of hydrogen and diesel fuel mixture, calculated using equation (30).

(30) $${LHV}_{mix}=y_{H_{2}}.{LHV}_{H_{2}}+y_{diesel}.{LHV}_{diesel}$$

Taking the above equations into account, the energy equation is written as equation (31).

(31) $$m_{t}.c_{v,i}.\left(T_{i}-T_{i-1}\right)=W_{combustion,i}+Q_{h,i}+Q_{i}$$

By solving the energy equation in conjunction with the pressure equation, represented as equation (32) and derived from the equation of state, the temperature and pressure values at the end of combustion (EOC) are determined.

(32) $$P_{i}=\left(\frac{V_{i-1}}{V_{i}}\right).\left(\frac{T_{i}}{T_{i-1}}\right){.P}_{i-1}$$

It is worth mentioning that the specific heat capacity at constant volume ($c_{v}$) is a critical parameter in single-zone models. Accurate, temperature- and composition-dependent values of $c_{v}$ are essential for precise temperature predictions, energy balance calculations, and combustion modeling. This study considers the variations of $c_{v}$ with temperature and composition throughout the combustion process.

#### 2.2.5 Polytropic expansion

The temperature and pressure at the end of the expansion stroke (EOV) are determined using the polytropic equations developed during the compression stroke. At this stage, the term $\left(P-P_{m}\right)\text{,}$ in equation (14), is replaced with $\frac{\left(\left(P_{evo}-P_{iso}\right)+\left(p_{evo}-P_{iso}\right)\right)}{2}$, which represents the average value of this pressure difference. Here, $P_{iso}$ denotes the pressure under isentropic conditions. Consequently, the work ($W_{expansion}$), heat transfer ($Q_{expansion}$), and polytropic exponent during the expansion stage ($K_{e}$) are calculated. The net work ($W_{net}$), Indicated Mean Effective Pressure (IMEP), and thermal efficiency ($\eta_{th}$) are then derived from equations (33), (34), (35)).

(33) $$W_{net}=W_{expansion}+W_{combustion}-W_{compresion}-W_{soi-soc}$$

(34) $$IMEP=\frac{W_{net}}{V_{d}}$$

(35) $$\eta_{th}=\frac{W_{net}}{Q_{ht}}$$

$Q_{ht}$ is the total heat release due to fuel combustion and is obtained from equation (36).

(36) $$Q_{ht}=\eta_{c}.m_{f}.{LHV}_{mix}$$

**Fig. 1:**
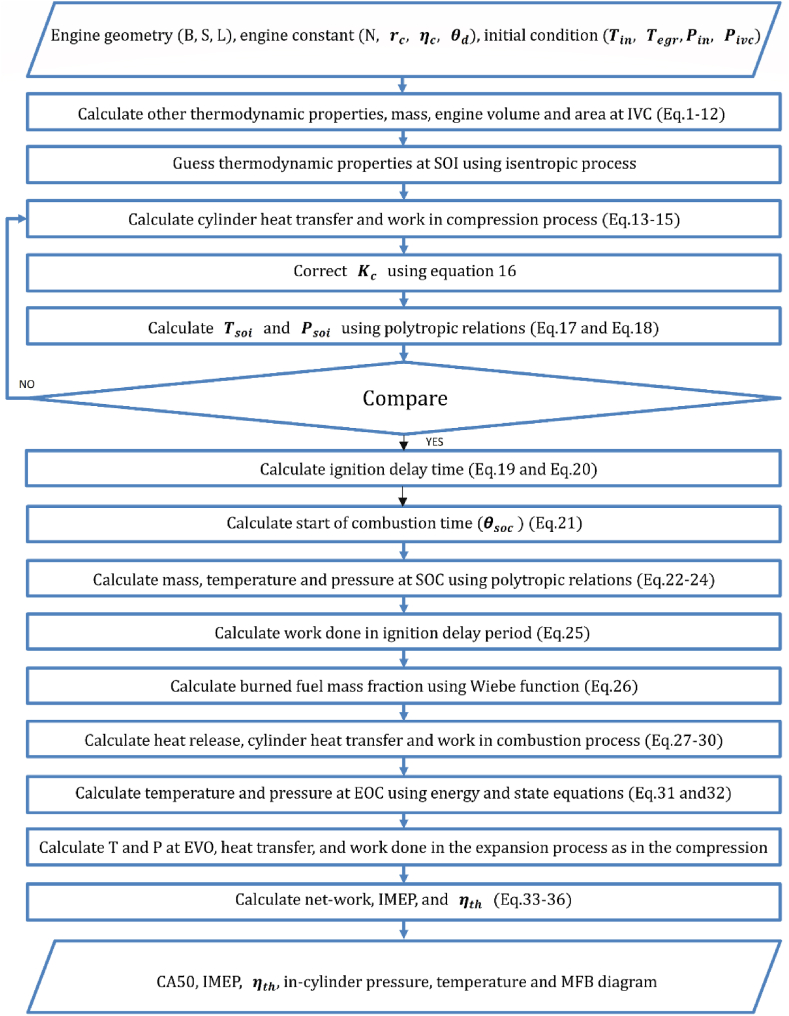
Flowchart of thermodynamic model of hydrogen/diesel premixed charge compression ignition engine.

The flowchart of the PCCI combustion model is shown in Fig. 1.

## 3 Validation of the model and discussion

There are few experimental studies on the use of hydrogen as a fuel in dual-fuel PCCI combustion. In this study, experimental data from Park et al. [44] were utilized for validation purposes. The engine analyzed in this simulation is a single-cylinder, direct injection, compression ignition engine, with specifications detailed in Table 1 [44]. This engine features a common rail injection system, a single-hole compressed natural gas (CNG) injector for hydrogen injection, and an EGR cooler. Based on the operating conditions outlined in Table 2 [44,52], four modes with varying hydrogen contents of 0 %, 30 %, 50 %, and 70 % were employed to validate the model. To maintain CA50 at 2.4 CAD aTDC, the EGR rate was adjusted across the different modes.

**Table 1:** Single cylinder engine specifications.

Parameter	Value
Bore (mm)	84
Stroke (mm)	90
Compression ratio	16:1
Displacement (L)	0.498
IVC (CAD aTDC)	−152
EVO (CAD aTDC)	126

**Table 2:** Operating conditions for validating the single-cylinder premixed charge compression ignition engine.

Parameters	%H2%DIESEL	
Diesel Injected Fuel (mg/Inje)	Case1(0%H2)	14.1
	Case2(30%H2)	9.8
	Case3(50%H2)	7
	Case4(70%H2)	4.2
(SOI)-(EGR)-$(\theta_{d}$)-(speed) (CAD aTDC-%- CAD-RPM)	Case1	(-32)-(43)-(17)-(1200)
	Case2	(-32)-(37)-(12.35)-(1200)
	Case3	(-32)-(32)-(9.16)-(1200)
	Case4	(-32)-(25)-(7.87)-(1200)

**Fig. 2:**
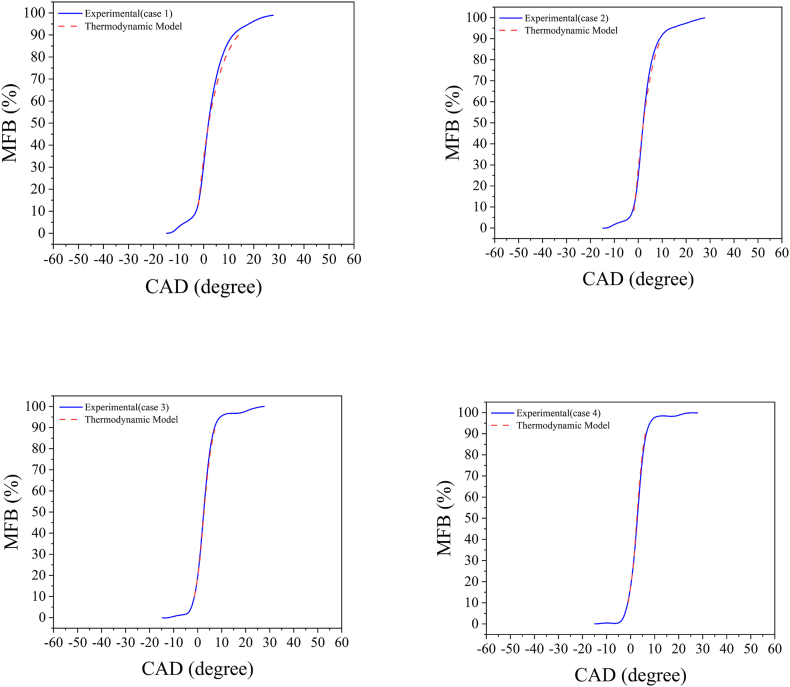
Comparison of the mass fraction burned diagrams from the thermodynamic model with experimental results (case 1- case 4).

In this validation, the plots of the burned fuel mass fraction, CA50, and IMEP model have been compared with the corresponding experimental values. The results of the burned fuel mass fraction plots are presented in Fig. 2.

As illustrated by the graphs, there is a good agreement between the experimental and simulated MFB results across all four operating modes. Accurate prediction of MFB is crucial in engine simulations as it significantly impacts the combustion phase, particularly CA50, and overall engine performance. Table 3 provides a comparison of the predicted CA50 location and IMEP with the experimental values.

**Table 3:** Comparison of the CA50 and indicated mean effective pressure results from the thermodynamic model with experimental results.

Case	1	2	3	4
Measured CA50 (CAD aTDC)	2.4	2.4	2.4	2.4
Predicted CA50 (CAD aTDC)	3	2.6	2.5	2.3
Measured IMEP (bar)	4.38	4.46	4.67	4.71
Predicted IMEP (bar)	4.386	4.4	4.682	4.75

This table indicates that the average predicted error for CA50 across all four operating conditions is 0.25 CAD. The predicted IMEP values for cases 1, 2, 3, and 4 are 4.386, 4.4, 4.682, and 4.75 bar, respectively. When compared to the experimental values, the average predicted error for IMEP across all cases is 0.043 bar. At a fixed CA50, increasing the hydrogen content results in a higher IMEP due to an increased heat release rate near TDC [53,54], which the model accurately captures. Consequently, the presented thermodynamic model demonstrates a strong capability to predict the combustion phase and performance of the dual-fuel PCCI engine.

It is important to note that, like all thermodynamic models, the presented model does not account for the effects of turbulence, temperature gradients at the cylinder wall, and low-temperature regions such as the crevice area where combustion probability is low. These factors can lead to inaccuracies in predicting IMEP and CA50. Therefore, some discrepancy between experimental and simulation results is inevitable in thermodynamic models. However, considering the aforementioned results, it can be concluded that the presented model has excellent potential for predicting the combustion and performance characteristics of dual-fuel engines and can be further developed for PCCI combustion control applications.

## 4 Verification of the model by CFD results and discussion

Due to the lack of detailed experimental data from the combustion state, such as in-cylinder temperature and pressure plots, which are essential for better validation and analysis of hydrogen/diesel dual-fuel PCCI combustion, the results of the thermodynamic model in this section are verified against the results of the 3D-CFD model. In previous work [17], the 1.9L GM PCCI engine was simulated and validated against experimental data using CONVERGE™. CONVERGE™, with its various sub-models and fully automated meshing process, enables the simulation of engines with complex geometries. The sub-models used in the CFD simulation, as well as the details and method of validation, are mentioned in Ref. [17]. The 1.9L GM engine is a single-cylinder version of a four-cylinder engine, with specifications shown in Table 4 [17]. The engine features a common rail injection system, a turbocharger with variable geometry (VGT), and variable actuator swirl. The experimental data used by Brakora and Reitz [55] for the PCCI engine at the University of Wisconsin were employed for validation. In this study, eight operating conditions were considered for verification of the PCCI engine, as listed in Table 5. In these conditions, the diesel injection quantity per cycle has been adjusted according to the hydrogen amount, ensuring that the total energy input to the engine remains constant at 630 J for cases A-G and 1000 J for case H. The plots of in-cylinder temperature and pressure, MFB curve, CA50, IMEP, and thermal efficiency of the model have been compared with the corresponding CFD model values.

**Table 4:** 1.9L GM engine specifications.

Parameter	Value
Bore (mm)	82
Stroke (mm)	90.4
Compression ratio	16.6:1
Displacement (L)	0.474
IVC (CAD aTDC)	−132
EVO (CAD aTDC)	112

**Table 5:** Operating conditions for the verification of the single-cylinder 1.9L GM premixed charge compression ignition engine.

Parameters	Case A	Case B	Case C	Case D	Case E	Case F	Case G	Case H
Hydrogen content (%)	0	10	30	30	50	30	30	70
Diesel fuel (mg/inj)	14.06	12.65	9.84	9.84	7	9.84	9.84	6.68
SOI (CAD aTDC)	−22	−22	−22	−22	−22	−30	−22	−22
EGR (%)	67	67	67	30	20	30	30	55
Engine speed (RPM)	2000	2000	2000	2000	2000	2000	1500	2000

**Fig. 3:**
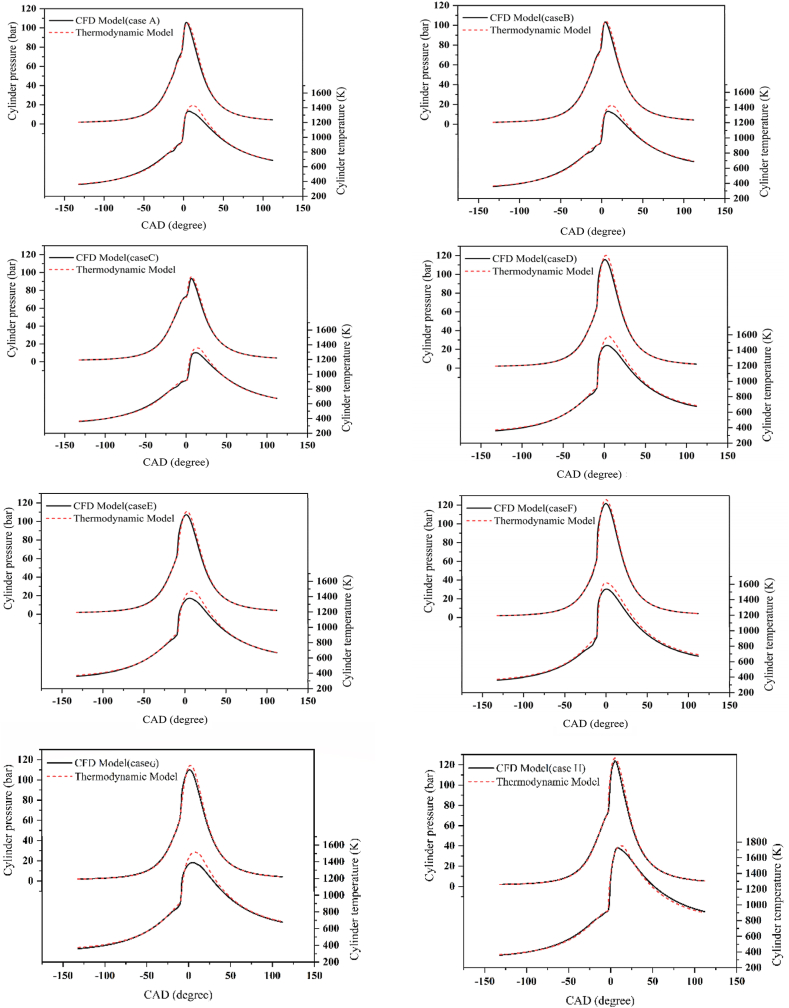
Comparison of in-cylinder pressure and temperature diagrams from the thermodynamic model with CFD results (case A-case H).

The comparison results of in-cylinder pressure and temperature between the CFD model and the thermodynamic model are presented in Fig. 3. The data indicates a strong correlation between the CFD results and the model predictions for in-cylinder pressure during the compression, combustion, and expansion phases, as well as for in-cylinder temperature during the compression and expansion phases, across all eight cases. The maximum percentage difference in peak pressure between the CFD and thermodynamic model results is 3.85 %, observed in case G. The thermodynamic model, being a single-zone model, tends to over-predict peak pressures and in-cylinder temperatures compared to a multi-zone model like the CFD model. This discrepancy is clearly illustrated in Fig. 3. The over-prediction in single-zone models arises from simplistic and unrealistic assumptions, such as uniform temperature and pressure within the combustion chamber. In reality, inhomogeneities within the cylinder lead to varying temperatures and pressures in different regions, such as near the cylinder wall, crevices, and mixed and unmixed zones.

The CFD model accounts for flow turbulence, as well as temperature and pressure gradients in these regions, resulting in predictions that are closer to the actual operational performance of the engine. However, this increased accuracy necessitates solving complex fluid dynamics equations, which requires significant computational time and resources. As demonstrated, the presented model can effectively simulate the engine's temperature, pressure, and combustion phase with minimal acceptable error and in a significantly reduced time frame.

To investigate the impact of adding hydrogen to the PCCI engine, cases A-C are considered. When hydrogen is introduced to the engine, while keeping other parameters constant, the peak pressure and temperature decrease. This is because the addition of hydrogen reduces the amount of diesel fuel input, maintaining a constant total energy content, which subsequently lowers the low-temperature heat release (LTHR) of the diesel fuel. Furthermore, the introduction of hydrogen increases the consumption of hydroxyl radicals in low-temperature reactions within the LTHR region associated with diesel fuel [54,56]. This causes the temperature required for the auto-ignition of pre-mixed mixtures to be reached later, which in turn reduces the overall heat release rate and retards the combustion phase. These factors collectively lead to a decrease in peak temperature and pressure. Fig. 3 clearly illustrates that the presented model accurately captures this reduction in temperature and pressure due to the addition of hydrogen.

By comparing case D with case C, the effect of EGR can be examined. Increasing the EGR percentage reduces the temperature and pressure inside the combustion chamber due to its high heat capacity. Additionally, it decreases the amount of oxygen available in the combustion chamber, which reduces the total heat released and further lowers the temperature and pressure [17]. The presented model accurately demonstrates this reduction in temperature and pressure due to the increased EGR percentage.

The effect of injection timing on the temperature and pressure inside the cylinder can be analyzed by comparing case D with case F. By examining the pressure and temperature graphs for these two cases, it is observed that early injection timing increases the temperature and pressure inside the chamber. The earlier the fuel is injected, the longer the ignition delay period [17]. With an increased ignition delay, there is more time for better mixing of fuel and air, resulting in more fuel burning in the premixed combustion phase, which increases the peak pressure and temperature inside the combustion chamber. The presented model also accurately demonstrates this change in temperature and pressure due to variations in fuel injection timing.

To analyze the impact of engine speed on the temperature and pressure inside the cylinder, cases D and G are considered. As observed, a decrease in engine speed results in lower in-cylinder temperature and pressure. The slower engine speed allows the combustion process to take longer to complete. This extended combustion period leads to a more gradual increase in cylinder pressure and provides additional time for heat transfer. These factors collectively reduce the peak pressure and temperature. The presented model effectively demonstrates the influence of engine speed on temperature and pressure.

**Fig. 4:**
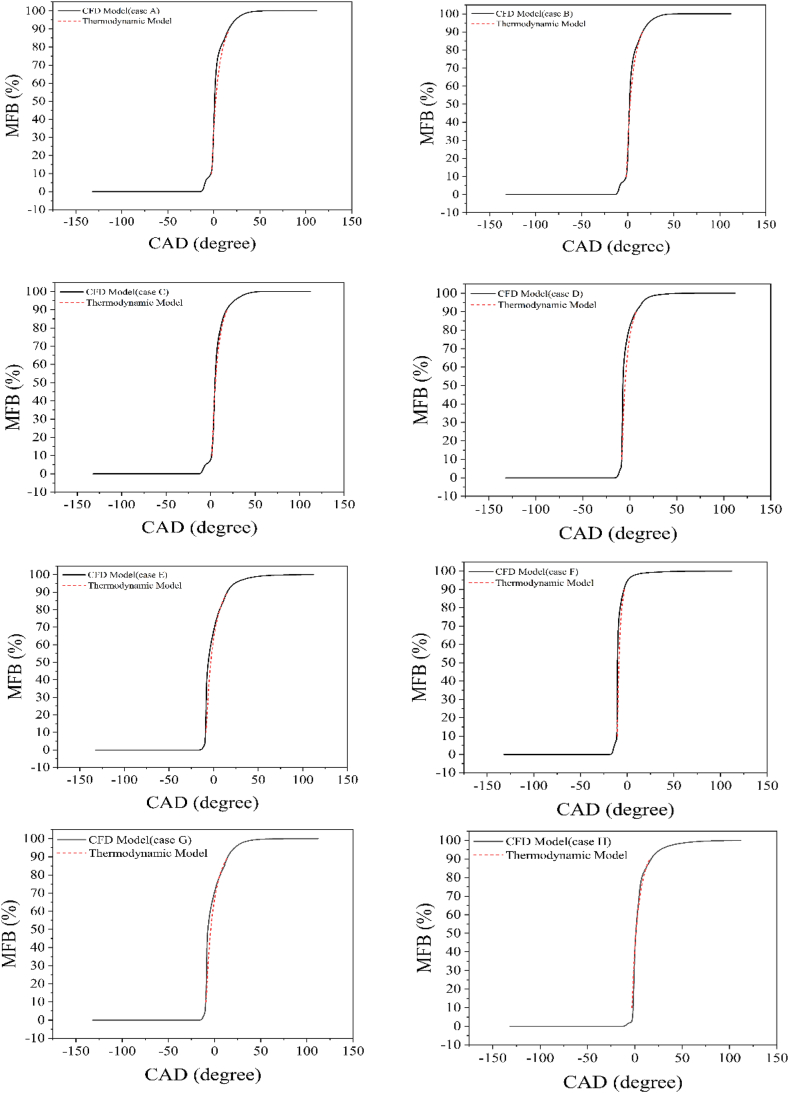
Comparison of the mass fraction burned diagrams from the thermodynamic model with CFD results (case A-case H).

Fig. 4 illustrates the MFB curves of the CFD and thermodynamic models. The single-Wiebe function accurately predicts the mass fraction burned of the CFD model from 10 % to 90 % of the burnt fuel mass. It is noteworthy that assuming 100 % combustion for the single-Wiebe function makes it challenging to simultaneously match both the timing and the value of the peak pressure. However, this can be achieved with less than 100 % combustion [40]. Considering the assumed Wiebe parameters, the average error between the peak pressure timing of the thermodynamic model and the CFD model is 0.6 CAD. In the CFD model, CA50 values are 0.95, 1.95, 5.05, −7.35, −5.95, −10.25, −7.17, and 1.15 CAD aTDC for cases A, B, C, D, E, F, G, and H respectively, while the corresponding values for the thermodynamic model are 2, 2.5, 5.4, −5.5, −3.4, −9.5, −4, and 1.28 CAD aTDC. The average error of CA50 between the thermodynamic model and the CFD model is 1.28 CAD. Therefore, the presented model demonstrates a high degree of precision in predicting peak pressure timing and CA50.

Increasing the amount of hydrogen and the EGR percentage delays the combustion phase, while early fuel injection advances it. Additionally, reducing engine speed extends the combustion duration. As illustrated in Fig. 4, the presented model accurately predicts all these changes in the combustion phase of the PCCI engine.

**Fig. 5:**
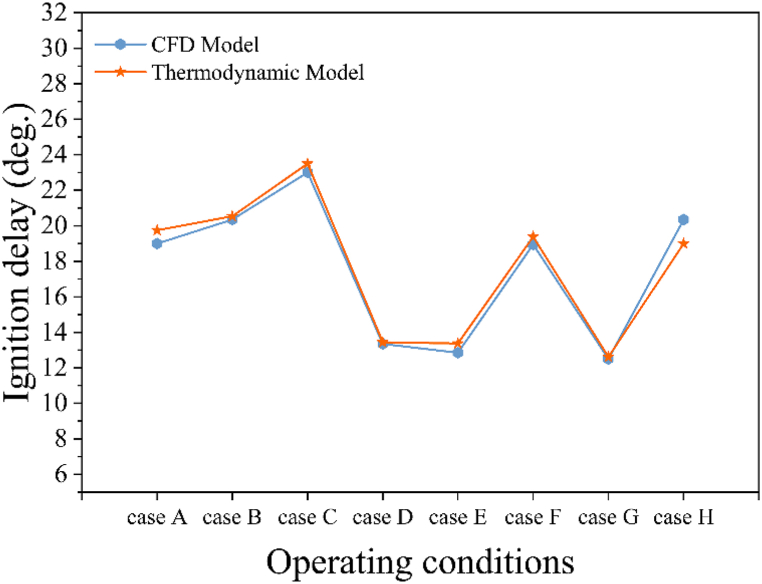
Comparison of the ignition delay periods between thermodynamic and CFD models.

In addition to analyzing and comparing the MFB curves, it is essential to evaluate the accuracy of the ignition delay model and compare the heat release rate (HRR) curves of both thermodynamic and CFD models for a comprehensive assessment of the engine combustion phase. Fig. 5 illustrates the comparison between the predicted ignition delay periods for the operating conditions specified in Table 4 and those obtained from the CFD model. The figure reveals that the maximum discrepancy between the ignition delay periods predicted by the model and those from the CFD analysis occurs in case H, with a value of 1.35 CAD, and the average discrepancy is only 0.5 CAD. Consequently, the proposed model demonstrates a high capability in accurately predicting ignition delay across various operating conditions.

**Fig. 6:**
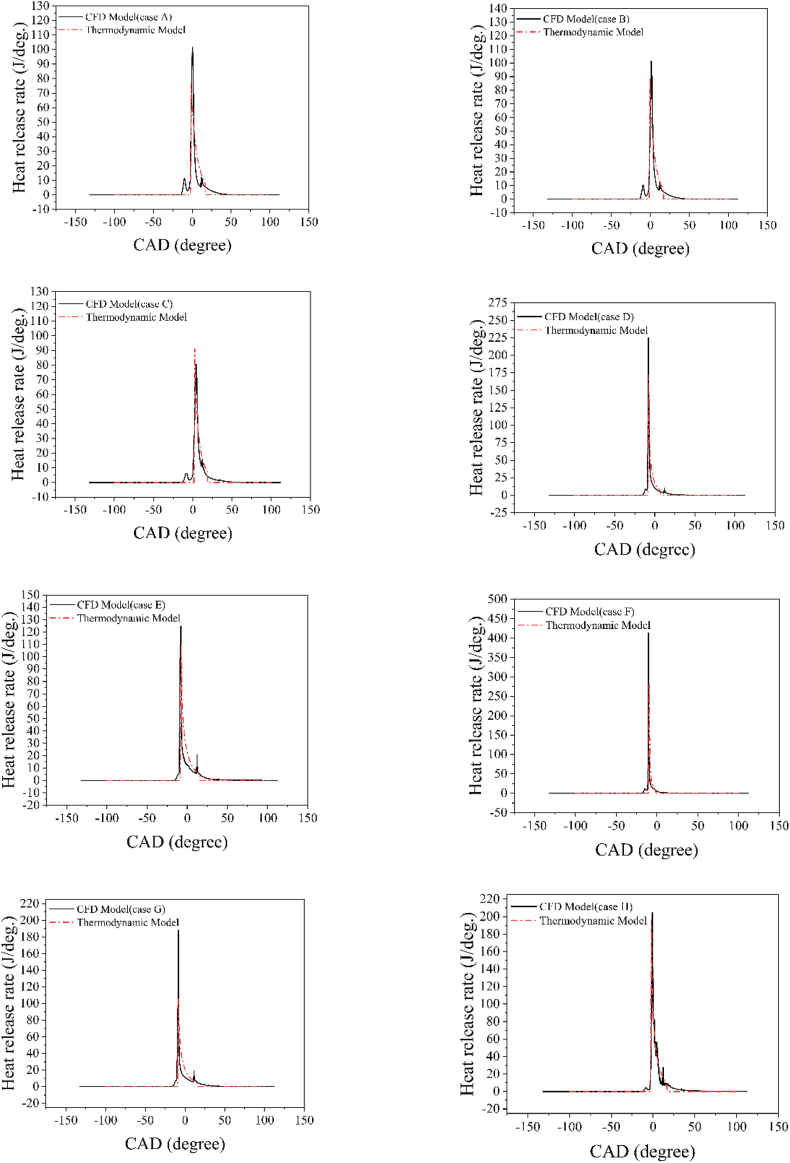
Comparison of the heat release rate diagrams from the thermodynamic model with CFD results (case A-case H).

Fig. 6 illustrates the heat release rate diagrams obtained from both the thermodynamic and CFD models. Despite the inherent complexities of combustion dynamics, which can be influenced by variables such as fuel properties, fuel-air mixture quality, and turbulence, Fig. 6 demonstrates that the proposed single Wiebe model accurately predicts the heat release rate trend. The peak positions of the heat release rate for the CFD model are 0.35, 1.25, 4.25, −8, −8, −10.35, −8.4, and −1 CAD aTDC for cases A to H, respectively. In comparison, the corresponding values for the thermodynamic model are −1.3, −0.5, 2.5, −7.6, −7.6, −9.6, −8.4, and −2 CAD aTDC. The average relative error of the peak heat release rate between the thermodynamic and CFD models is 0.835 CAD. Therefore, considering the MFB diagrams, ignition delay, and heat release rate diagrams, the presented ignition delay correlation and single Wiebe function with the assumed parameters can predict the combustion phase of the PCCI engine with high accuracy.

**Fig. 7:**
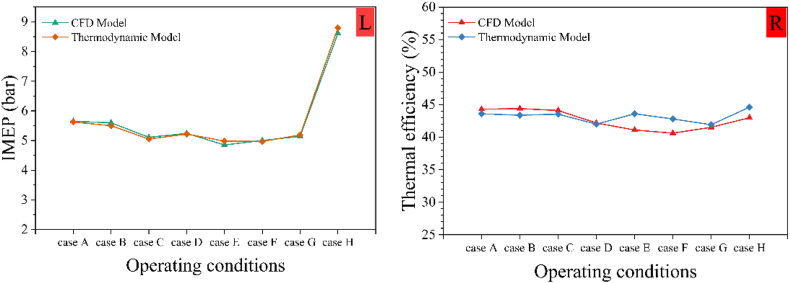
Comparison of the indicated mean effective pressure (L) and thermal efficiency (R) results between thermodynamic and CFD models.

Fig. 7 compares the IMEP and thermal efficiency of the CFD and thermodynamic models under various operating conditions. The IMEP values using the CFD model are 5.65, 5.6, 5.11, 5.24, 4.85, 5, 5.14, and 8.615 bar for cases A, B, C, D, E, F, G, and H, respectively. The corresponding values for the thermodynamic model are 5.625, 5.5, 5.05, 5.22, 4.98, 4.97, 5.187, and 8.8 bar, with a maximum difference of 2.7 %.

The thermal efficiency values for the CFD model are 44.3 %, 44.4 %, 44.1 %, 42.18 %, 41.1 %, 40.62 %, 41.52 %, and 43 % for cases A, B, C, D, E, F, G, and H, respectively. The corresponding values for the thermodynamic model are 43.6 %, 43.37 %, 43.54 %, 42 %, 43.6 %, 42.8 %, 41.92 %, and 44.6 %, with an average error of 2.41 %.

As mentioned, increasing hydrogen reduces the temperature and heat release rate inside the cylinder, resulting in less cumulative heat release and consequently lowering the in-cylinder pressure and IMEP. Increasing EGR reduces the temperature inside the combustion chamber, thereby decreasing the pressure and IMEP. Early diesel fuel injection significantly advances the combustion phase, causing more combustion to occur before TDC, which reduces IMEP. Under similar conditions, reducing engine speed lowers the pressure inside the chamber and decreases IMEP. The presented model accurately predicts these IMEP changes due to variations in key PCCI engine parameters.

## 5 Conclusions

In recent years, hydrogen fuel has garnered significant attention due to its high environmental compatibility. Utilizing hydrogen fuel in low-emission, LTC engines can enhance engine efficiency while markedly reducing environmental pollutants. Developing thermodynamic models with low computational time and cost not only aids in the control and optimization of LTC engines fueled with hydrogen but also significantly facilitates the assessment of their applications in hybrid systems. This approach greatly contributes to the expansion of environmentally friendly systems. In this work, a simple thermodynamic-empirical model was developed to simulate the hydrogen/diesel dual-fuel PCCI engine from IVC to EVO using EES software. In this model, to enhance the accuracy of engine performance predictions, the appropriate polytropic exponent for each operating condition is calculated using an iterative method. Additionally, heat transfer correlations are considered throughout all engine processes.

For validating the results of the presented physics-based model, four engine operation modes were considered, and the MFB curves, CA50, and IMEP model were compared with experimental results. In the second part, to further evaluate the proposed model, the CFD simulation results of the 1.9L GM PCCI engine were used to verify the thermodynamic model results. For verification, the pressure and temperature plots, MFB plots, IMEP values, and thermal efficiency of the CFD model were compared with those of the thermodynamic model. The following conclusions are derived from the study:

1. The validation results showed that the average predicted error of CA50 for all four operating conditions was 0.25 CAD, and the average predicted error related to IMEP for all four cases was 0.043 bar. This indicates that the presented model has a very good ability to predict the PCCI combustion phase.
2. Considering the verification results, the highest percentage difference in peak pressure between the CFD and thermodynamic model results was 3.85 %. The average error in the peak pressure timing between the thermodynamic model and the CFD model results was 0.6 CAD.
3. With the assumed Wiebe function parameters, the single-Wiebe function could accurately predict the mass fraction burned of the CFD model from 10 % to 90 % of the burnt fuel mass. The average error of the CA50 between the thermodynamic model and the CFD model results was 1.28 CAD.
4. The highest percentage difference in IMEP between the CFD model and the thermodynamic model results was 2.7 %, and the average error in thermal efficiency between the CFD model and the thermodynamic model results was 2.41 %.
5. Comparison of the CFD model results with the thermodynamic model results shows that despite the simplicity of the presented single-zone model, its results are very close to those of the CFD model. It is clear that the CFD model, due to its characteristics, provides more accurate and reliable results. However, the comparison showed that the presented model provides sufficiently accurate and reliable results without the complexity and high cost of the CFD model.
6. The study of the impact of changes in key engine parameters, such as hydrogen quantity, EGR percentage, injection timing, and engine speed on its performance, showed that the presented model correctly reflects the trend of these parameter changes on engine performance, in accordance with CFD results.

The validation and verification results show that by developing the model for the intake and exhaust processes and simulating the engine cycle-to-cycle, it can be used for control applications of dual-fuel PCCI combustion. In future work, the presented model can be improved by using a dual-Wiebe function, developing it into a two-zone model to predict the combustion phase more accurately, and providing a better model for predicting the start of combustion to investigate other bio-derived fuels.

## CRediT authorship contribution statement

**Ghasem Jalivar:** Writing – original draft, Validation, Methodology, Investigation, Formal analysis. **Elaheh Neshat:** Writing – review & editing, Supervision, Methodology.

## Data availability statement

Data will be made available on request.

## Declaration of competing interest

The authors declare that they have no known competing financial interests or personal relationships that could have appeared to influence the work reported in this paper.

## Appendix A Model sub-equations

### A.1. Equivalence ratio

In Equation (12), ∅ is the equivalence ratio. The equivalence ratio is defined as the ratio of stoichiometric air-to-fuel to actual air-to-fuel and is obtained from relations A.1.1-A.1.3.

(A.1.1) $$\emptyset =\frac{{AF}_{st}}{{AF}_{ac}}$$

(A.1.2) $${AF}_{st}=\frac{\left(x+y⁄4\right)+b/2)4.76{MW}_{air}}{{MW}_{f}}$$

(A.1.3) $${AF}_{ac}=\frac{m_{air}}{m_{f}}$$

Where ${AF}_{st}$ and ${AF}_{ac}$ are stoichiometric and actual air to fuel ratio respectively, ${MW}_{air}$ is the molecular mass of air and ${MW}_{f}$ is the molecular mass of fuel.

### A.2. Gas velocity characteristic in Woschni correlation

In Equation (14), w is the gas velocity characteristic, calculated using the following equation.

(A.2.1) $$w=C_{1}.S_{p}+\frac{C_{2}.V_{d}.T_{r}}{6V_{r}.P_{r}}.\left(P-P_{m}\right)$$

Where $S_{p}$ is the average piston speed, $T_{r}$, $P_{r}$, and $V_{r}$ are the temperature, pressure, and volume at IVC. ($C_{1}$ = 2.28) (adjustable by engine specifications) and ($C_{2}$ = 0.324). P and $P_{m}$ are the instantaneous and motoring pressures, respectively. The second term on the right of the gas velocity equation during compression is zero due to no combustion. $P_{m}$ is obtained as follows.

(A.2.2) $$P_{m}=P_{ivc}{.\left(\frac{V_{ivc}}{V_{i}}\right)}^{K_{c}}$$

### A.3. Weibe function parameters

In Equation (26), $a^{\prime }$ and $m^{\prime }$ are Weibe function parameters. $a^{\prime }$ indicates the combustion efficiency factor, which is obtained from the following equation based on the definition of the end of combustion [57]:

(A.3.1) $$a^{\prime }=\ln\;\left(1-x_{b,eoc}\right)$$

*m'*, the shape factor, indicates the shape of the burning curve and is calculated using the following equation [58].

(A.3.2) $$\left(\theta_{90}-\theta_{10}\right)=\left(\theta_{99.9}-\theta_{0}\right).\left(\left({\left(\frac{\ln\left(0.1\right)}{\ln\left(0.001\right)}\right)}^{\frac{1}{m^{\prime }+1}}-{\left(\frac{\ln\left(0.9\right)}{\ln\left(0.001\right)}\right)}^{\frac{1}{m^{\prime }+1}}\right)\right)$$

Where $\theta_{10}$, $\theta_{90}$ and $\theta_{99.9}$ are the angles corresponding to 10 %, 90 %, and 99.9 % of the burned fuel mass, respectively. $\theta_{0}$ is the start of combustion, which in this study is considered to be 2 % of the maximum heat release rate [59]. Additionally, $\left(\theta_{90}-\theta_{10}\right)$ is considered the combustion period for simulation, and $\left(\theta_{99.9}-\theta_{0}\right)$ is considered the complete combustion period [58].

## References

[1] Bari S., Hossain S.N. (2013). Waste heat recovery from a diesel engine using shell and tube heat exchanger. Appl. Therm. Eng..

[2] Shadidi B., Najafi G., Yusaf T. (2021). A review of hydrogen as a fuel in internal combustion engines. Energies.

[3] Imtenan S. (2014). Impact of low temperature combustion attaining strategies on diesel engine emissions for diesel and biodiesels: a review. Energy Convers. Manag..

[4] Meldahl L.L. (2021). Comparing Norway's and the Netherland's Roles in the European Hydrogen Transition.

[5] Formica M., Frigo S., Gabbrielli R. (2016). Development of a new steady state zero-dimensional simulation model for woody biomass gasification in a full scale plant. Energy Convers. Manag..

[6] Alagumalai A. (2023). Machine learning in biohydrogen production: a review. Biofuel Research Journal.

[7] Saghir M., Rehan M., Nizami A.-S. (2018). Gasification for Low-Grade Feedstock.

[8] Gao Y. (2023). Syngas production from biomass gasification: influences of feedstock properties, reactor type, and reaction parameters. ACS Omega.

[9] Makepa D.C., Chihobo C.H. (2024). Sustainable pathways for biomass production and utilization in carbon capture and storage—a review. Biomass Conversion and Biorefinery.

[10] Kumar M.S., Muniyappan M., Selvan S.A. (2024). Experimental and CFD analysis on the impact of hydrogen as fuel on the behavior of a passenger car gasoline direct injection engine. J. Energy Inst..

[11] Hosseini S.H. (2023). Use of hydrogen in dual-fuel diesel engines. Prog. Energy Combust. Sci..

[12] Özyalcin C. (2024). Exhaust gas aftertreatment to minimize NOX emissions from hydrogen-fueled internal combustion engines. Appl. Energy.

[13] Yip H.L. (2019). A review of hydrogen direct injection for internal combustion engines: towards carbon-free combustion. applied sciences.

[14] Jilakara S. (2015). An experimental study of turbocharged hydrogen fuelled internal combustion engine. SAE International Journal of Engines.

[15] Reijnders J., Boot M., de Goey P. (2016). Impact of aromaticity and cetane number on the soot-NOx trade-off in conventional and low temperature combustion. Fuel.

[16] Gupta S.K., Krishnasamy A. (2024). A relative comparison of HCCI, PCCI, and RCCI combustion strategies: an alternative fuels perspective. Int. J. Engine Res..

[17] Jalivar G., Saray R.K., Neshat E. (2023). Investigation of PCCI combustion and emissions of biodiesel fuel at low load conditions using design of experiment (DOE). J. Therm. Anal. Calorim..

[18] Elkelawy M. (2022). A critical review of the performance, combustion, and Emissions characteristics of PCCI engine controlled by injection strategy and fuel properties. Journal of Engineering Research.

[19] Alemayehu G. (2022). Operating parameters optimization for lower emissions in diesel engine with PCCI-DI mode using Taguchi and grey relational analysis. Heliyon.

[20] Bidarvatan M. (2014). Cycle-to-cycle modeling and sliding mode control of blended-fuel HCCI engine. Control Eng. Pract..

[21] Tang J., Zhu G.G., Men Y. (2022). Review of engine control-oriented combustion models. Int. J. Engine Res..

[22] Choi Y., Chen J.-Y. (2005). Fast prediction of start-of-combustion in HCCI with combined artificial neural networks and ignition delay model. Proc. Combust. Inst..

[23] Janakiraman V.M., Nguyen X., Assanis D. (2013). Nonlinear identification of a gasoline HCCI engine using neural networks coupled with principal component analysis. Appl. Soft Comput..

[24] Shamekhi A.-M., Shamekhi A.H. (2015). A new approach in improvement of mean value models for spark ignition engines using neural networks. Expert Syst. Appl..

[25] Bastawissi H. (2010). Detailed 3D-CFD/chemistry of CNG-hydrogen blend in HCCI engine. SAE Technical Paper.

[26] Hessel R.P. (2008). Modeling iso-octane HCCI using CFD with multi-zone detailed chemistry; comparison to detailed speciation data over a range of lean equivalence ratios. SAE Technical Paper.

[27] Neshat E., Saray R.K. (2014). Development of a new multi zone model for prediction of HCCI (homogenous charge compression ignition) engine combustion, performance and emission characteristics. Energy.

[28] Aceves S.M. (2000). A multi-zone model for prediction of HCCI combustion and emissions. SAE Trans..

[29] Campbell M.F., Davidson D.F., Hanson R.K. (2016). Scaling relation for high-temperature biodiesel surrogate ignition delay times. Fuel.

[30] Amjad A. (2011). Availability analysis of n-heptane and natural gas blends combustion in HCCI engines. Energy.

[31] Jia M. (2011). The effect of injection timing and intake valve close timing on performance and emissions of diesel PCCI engine with a full engine cycle CFD simulation. Appl. Energy.

[32] Pandey S., Bhurat S., Chintala V. (2019). Combustion and emissions behaviour assessment of a partially premixed charge compression ignition (PCCI) engine with diesel and fumigated ethanol. Energy Proc..

[33] Andrae J.C., Head R. (2009). HCCI experiments with gasoline surrogate fuels modeled by a semidetailed chemical kinetic model. Combust. Flame.

[34] Yamasaki Y. (2019). Simple combustion model for a diesel engine with multiple fuel injections. Int. J. Engine Res..

[35] Bissoli M. (2016). A new predictive multi-zone model for HCCI engine combustion. Appl. Energy.

[36] Visakhamoorthy S. (2012). Numerical study of a homogeneous charge compression ignition (HCCI) engine fueled with biogas. Appl. Energy.

[37] Barroso Raya G. (2006).

[38] Shaver G.M., Roelle M., Gerdes J.C. (2006). 2006 American Control Conference.

[39] Yasar H. (2008). Double-Wiebe function: an approach for single-zone HCCI engine modeling. Appl. Therm. Eng..

[40] Ravi N. (2009). Model-based control of HCCI engines using exhaust recompression. IEEE Trans. Control Syst. Technol..

[41] Namar M.M., Jahanian O. (2019). Energy and exergy analysis of a hydrogen-fueled HCCI engine. J. Therm. Anal. Calorim..

[42] Xiao J. (2024). Thermodynamic and heat transfer models for refueling hydrogen vehicles: formulation, validation and application. Int. J. Hydrogen Energy.

[43] Caton J.A. (2015).

[44] Park H., Kim J., Bae C. (2015). Effects of hydrogen ratio and EGR on combustion and emissions in a hydrogen/diesel dual-fuel PCCI engine. SAE Technical Paper.

[45] Heywood J.B. (1988).

[46] Chang J. (2004). New heat transfer correlation for an HCCI engine derived from measurements of instantaneous surface heat flux. SAE Trans..

[47] Assanis D.N. (2003). A predictive ignition delay correlation under steady-state and transient operation of a direct injection diesel engine. J. Eng. Gas Turbines Power.

[48] Van Alstine D.G. (2013). Control-oriented premixed charge compression ignition combustion timing model for a diesel engine utilizing flexible intake valve modulation. Int. J. Engine Res..

[49] Zheng M. (2008). Biodiesel engine performance and emissions in low temperature combustion. Fuel.

[50] Kirgina M.V. (2019). Calculation method for prediction of the cetane index of blended diesel fuels. Petroleum & Coal.

[51] Vibe I. (1970).

[52] Kim J., Bae C. (2016). An investigation on the effects of late intake valve closing and exhaust gas recirculation in a single-cylinder research diesel engine in the low-load condition. Proc. Inst. Mech. Eng. - Part D J. Automob. Eng..

[53] Guo H., Neill W.S. (2013). The effect of hydrogen addition on combustion and emission characteristics of an n-heptane fuelled HCCI engine. Int. J. Hydrogen Energy.

[54] Shudo T., Yamada H. (2007). Hydrogen as an ignition-controlling agent for HCCI combustion engine by suppressing the low-temperature oxidation. Int. J. Hydrogen Energy.

[55] Brakora J., Reitz R.D. (2013). A comprehensive combustion model for biodiesel-fueled engine simulations.

[56] Guo H. (2011). An experimental study on the effect of hydrogen enrichment on diesel fueled HCCI combustion. Int. J. Hydrogen Energy.

[57] Ghojel J. (2010). Review of the development and applications of the Wiebe function: a tribute to the contribution of Ivan Wiebe to engine research. Int. J. Engine Res..

[58] Lindström F. (2005). An empirical SI combustion model using laminar burning velocity correlations. SAE Trans..

[59] Witt A. (1999).

